# Local penetration of doxorubicin via intrahepatic implantation of PLGA based doxorubicin-loaded implants

**DOI:** 10.1080/10717544.2019.1676842

**Published:** 2019-11-06

**Authors:** Li Gao, Qingshan Li, Jie Zhang, Yixin Huang, Lin Deng, Chenyang Li, Guangping Tai, Banfeng Ruan

**Affiliations:** a School of Food and Biological Engineering, Hefei University of Technology, Hefei, People’s Republic of China;; b Key Lab of Biofabrication of Anhui Higher Education Institution Centre for Advanced Biofabrication, Hefei University, Hefei, People’s Republic of China

**Keywords:** Doxorubicin, implants, UPLC-MS/MS, minipig, local penetration

## Abstract

Doxorubicin (DOX) is widely used in the chemotherapy of a wide range of cancers. However, intravenous administration of DOX causes toxicity to most major organs which limits its clinical application. DOX-loaded drug delivery system could provide a continuous sustained-release of drugs and enables high drug concentrations at the target site, while reducing systemic toxicity. Additionally, local chemotherapy with DOX may be a promising approach for lowering post-surgical recurrence of cancer. In this study, the sustained-release DOX-loaded implants were prepared by melt-molding method. The implants were characterized with regards to drug content uniformity, micromorphology and drug release profiles. Furthermore, differential scanning calorimetry (DSC) and Fourier transform infrared spectroscopy (FTIR) analyses were carried out to investigate the drug-excipient compatibility. To determine the local penetration of DOX in liver, the minipigs received intrahepatic implantation of DOX-loaded implants by abdominal surgery. UPLC-MS/MS method was used to detect the concentration of DOX in liver tissues. Our results suggested that DOX-loaded implants delivered high doses of drug at the implantation site for a prolonged period and provided valuable information for the future clinical applications of the DOX-loaded implants.

## 1. Introduction

Doxorubicin (DOX) is widely used in the chemotherapy of a wide range of cancers, including breast, bileducts, prostate, uterus, ovary, esophagus, stomach and liver tumors, childhood solid tumors, osteosarcomas and soft tissue sarcomas, Kaposi’s sarcoma, acute myeloblastic leukemia, lymphoblastic leukemia and Wilms tumor (Carvalho et al., 2009). As a frontline drug, DOX has been used for treating cancer for over 30 years and was regarded as one of the most potent FDA-approved chemotherapeutic drugs (Tacar et al., 2013). The mechanisms of DOX-mediated cell death include topoisomerase II poisoning, DNA adduct formation, oxidative stress and ceramide overproduction. Moreover, the intercalation of DOX between DNA bases has a direct effect on chromatin and results in cancer cells killing (Yang et al., 2014). However, the clinical application of DOX is limited by its multi-organ toxicities, especially cardiotoxicity (Pugazhendhi et al., 2018). DOX presents a dose-dependent and cumulative cardiotoxicity which is the most serious side-effect and affect the patients’ overall outcomes (Carvalho et al., 2014; Cappetta et al., 2018).

Local chemotherapy with polymer-based drug delivery systems provides a continuous sustained release of anticancer drugs and enables high drug concentrations at the target site for a prolonged time, while reducing systemic toxicity (Gao et al., 2019). Furthermore, the targeted chemotherapy has been considered as potential future solution for both prevention and treatment of locally recurrent cancers (Mahvi et al., 2018).

Local recurrence after surgical resection has negative effect on the survival time and quality of life for cancer patients (Mahvi et al., 2018). Local chemotherapy with polymer-based drug delivery systems can provide high, localized doses of chemotherapy at the site of the tumor resection for a prolonged period, therefore it is considered to be a promising approach for preventing recurrence (Saltzman & Fung, 1997; Wolinsky et al., 2012). The Gliadel^®^ (MGI Pharma/Easai Pharmaceuticals) is the most successful carmustine-loaded biodegradable polymer wafer used for the treatment of recurrent malignant glioma. In the clinical application, the Gliadel^®^ are deposited along the resection cavity following surgical excision of the primary brain tumor and has shown increased survival in patients (Wolinsky et al., 2012; Shapira-Furman et al., 2019).

The novel DOX-loaded drug delivery systems may be an alternative of conventional treatment where DOX can act directly on the tumors and enhance the efficacy of the drug, while lowering the systemic toxicity, such as liposomes, hydrogels and nanoparticles (Tacar et al., 2013; Gabizon et al., 2016; Fojtu et al., 2017). In this study, we prepared DOX-loaded implants using polylactic-co-glycolic acid (PLGA) as drug delivery carrier. Then the implants were characterized in terms of drug content uniformity, micromorphology, *in vitro* and *in vivo* drug release profiles, differential scanning calorimetry (DSC) and Fourier transform infrared spectroscopy (FTIR) analyses. To gain the insight into the local penetration of DOX in liver after intrahepatic implantation of DOX-loaded implants, we developed the accurate and reliable ultra high performance liquid chromatography-tandem mass spectrometry (UPLC-MS/MS) method to detect DOX concentration in liver tissues in the implantation site and around the implantation site.

## 2. Materials and methods

### 2.1. Reagents and animals

Doxorubicin hydrochloride (Lot:1050-C180201, purity ≥ 99.8%) was obtained from Zhejiang Hisun Pharmaceutical Co., Ltd. (Zhejiang, China). Glibenclamide was purchased from Sigma (St Louis, MO, USA). PLGA (75:25 lactide/glycolide; inherent viscosity 0.21 dL/g) was generously provided by Hefei Zhongren Science and Technoloty Co., Ltd. (Hefei, China). Polyethylene glycol 4000 (PEG 4000) was from Beijing Huiyou Chemical Co., Ltd. (Beijing, China). HPLC-grade formic acid was obtained from Tianjin Kermel Chemical Reagent Co., Ltd. (Tianjin, China). HPLC-grade methanol and acetonitrile were obtained from Tedia Co., Inc. (Fairfield, OH, USA). Ultra-pure water was obtained in a milli-Q system from Millipore (Milford, MA, USA).

The adult and healthy Guangxi Bama minipigs, half male and half female, were purchased from Taizhou miniature pig breeding base (Taizhou, China). The Wistar rats were purchased from Experimental Animal Center of Anhui Medical University. The animal experiments were complied with the ARRIVE guidelines and carried out in accordance with the U.K. Animals (Scientific Procedures) Act, 1986 and associated guidelines.

### 2.2. Preparation and characterization of DOX-loaded implants

#### 2.2.1. Preparation of DOX-loaded implants

The DOX-loaded implants were prepared by a melt-molding technique under sterile conditions. Briefly, the dry powders of DOX, PLGA and PEG 4000 were sieved separately through 120-mesh size sieve and thoroughly blended in a ratio of 5:13:2 (w/w/w). Then the homogenous mixture was dried under vaccum and heated at 110 °C for 10–12 min until it was completely melted. The resultant blend was put into the mold and further molded into cylindrical implants.

#### 2.2.2. Content uniformity of DOX-loaded implants

The drug content in the DOX-loaded implants was detected according to the method stated in the Chinese Pharmacopeia (China Pharmacopoeia Committee, 2015). Ten DOX-loaded implants were selected and weighed. Each implant sample was thoroughly dissolved in 10 mL of mobile phase. Then the resulting suspension was centrifuged at 12000 rpm for 10 min at 4 °C. Subsequently, an aliquot of the supernatant (10 μl) was analyzed by HPLC. The actual drug content of each implant was then calculated.

To evaluate the drug content uniformity, the acceptance value (AV) was calculated according to the instruction of the Chinese Pharmacopoeiaby by the formula: AV= |100 – $\bar{\text{X}}$| + 2.2S, where $\bar{\text{X}}$ is the mean of relative drug contents and S is the sample standard deviation. The maximum allowed AV value is set to 15.

#### 2.2.3. Scanning electron microscopy (SEM)

The surface and cross-section of the implants were observed using a JEOL JSM-6490LV scanning electron microscope (JEOL Co., Ltd., Tokyo, Japan) operating at a voltage of 20 kV. Prior to imaging, all samples were placed on metal sample holders and coated with gold for 90 s at 20 mA using JEOL JFC-1600 auto fine coater (JEOL Co., Ltd., Tokyo, Japan). The external and internal micromorphologies were imaged at ×600 magnification and ×1500 magnification, respectively.

#### 2.2.4. In vitro drug release assay

The *in vitro* release assay was performed using the rotating basket method on a dissolution apparatus. Fifty milligram of DOX-loaded implants were placed in 200 mL of release medium containing 0.01 mol/L Tris-HCl buffer (pH 4.0). The rotating speed of the basket was set at 120 rpm and the temperature of the release medium was maintained at (37 ± 0.5)°C. At different time points (0.5, 1, 2, 4, 5, 6, 8, 10, 12, 14, 24, 31 and 48 h post implantation), 5 mL of the sample was withdrawn, centrifuged at 12000 rpm for 10 min and stored at 4 °C until analysis. Then 5 mL of fresh release medium was immediately added back to the dissolution flask to maintain a constant sink condition. HPLC was used to quantify the DOX content of the implants. The measurement was performed in triplicate for each batch.

#### 2.2.5. In vivo drug release assay

To test the *in vivo* drug release of the DOX-loaded implants, an implant was surgically implanted into the liver of the 5 to 6-week-old female Wistar rats under sterile condition. At the predetermined time intervals (1, 3, 5, 7, 10, 15 and 25 days post implantation), the rats were euthanized by CO_2_ asphyxiation and the implants were retrieved, rinsed with deionized water, dried under vacuum and stored at 4 °C until analysis. Three animals were used at each time point. The amount of drug in the residual implant was determined by HPLC. The *in vivo* cumulative release percentage of DOX was calculated as follows:

$$\text{release percentage }(\%)=\frac{\text{initial DOX amount}-\text{residual DOX amount}}{\text{initial DOX amount}}\times 100\%.$$

#### 2.2.6. In vivo degradation study

To determine the *in vivo* dagradation of the PLGA based DOX-loaded implants, we implanted one blank implant (without DOX) into the liver of the rats. At the predetermined time points (7, 14, 28, 35, 42, 49 days post implantation), the rats were euthanized by CO_2_ asphyxiation and the blank implants were retrieved, rinsed with deionized water and dried under vacuum. Three rats were used at each time point. The percentage of weight loss was calculated according to the formula:

$$\text{weight loss }\left(\%\right)=\frac{\text{initial weight}-\text{residual weight}}{\text{initial weight}}\times 100\%.$$

#### 2.2.7. Differential scanning calorimetry (DSC) analysis

DSC analysis of the DOX-loaded implant was carried out with a TA instrument (Q2000, TA Instruments, New Castle, DE, USA). Samples (4-5 mg) of DOX-loaded implants, pure DOX, PLGA and PEG 4000 were sealed in aluminum pans and measured by DSC at a heating rate of 10 °C min^−1^ over a temperature range of 0–300 °C. High purity nitrogen was used as the purge gas at a flow rate of 50 mL/min.

#### 2.2.8. Fourier transform infrared spectroscopy (FTIR)

Infrared spectra of DOX-loaded implants, pure DOX, PLGA and PEG 4000 were generated in a FTIR spectrophotometer (Nicolet 6700, Thermo Nicolet corporation, USA) covering the range of 400–4000 cm^−1^. Measurements were carried out using the attenuated total reflectance technique. Each spectrum was a result of 32 scans with a resolution of 4 cm^−1^.

#### 2.2.9. The HPLC method for determination of DOX in the implants

The HPLC system (Shimadzu, Japan) was equipped with two LC-15C pumps, a SPD-15C essential UV detector and a CTO-15C essential column oven. A Hypersil BDS C6H5 column (250 mm × 4.6 mm, 5 μm particle size) was used as an analytical column and maintained at 25 °C in the column oven. The HPLC method for measuring DOX was in compliance with the instruction described in the Chinese Pharmacopeia (China Pharmacopoeia Committee, 2015). The mixture of sodium dodecylsulfate (SDS) solution (1.44 g of SDS and 0.68 mL of phosphoric acid were co-dissolved in 500 mL of ultra-pure water), acetonitrile and methanol was used as the mobile phase (500:500:60, v/v/v), and the flow rate was 1.0 mL/min. The injection volume was 10 μl and the absorbance was detected at 254 nm. The external standard method was used for quantitative analysis.

### 2.3. Local drug penetration

Fifteen minipigs weighing 12–15 kg were used in the experiment. All the animals were anesthetized intravenously with 3% sodium pentobarbital (1 mL/kg) and received abdominal surgery under anesthesia. The abdomen was disinfected with 75% ethanol and opened with an upper midline incision, then the DOX-loaded implants were inserted into the center of left lobe of liver at the dose of 2.3 mg/kg.

To determine the local drug penetration surrounding the implantation site, three minipigs were sacrificed at the scheduled time (day 1, 3, 7, 10 and 20 after treatment), the left lobe of liver was collected, preserved at −20 °C until analysis. After removing the drug residues in implantation site, serial liver sections of 2 mm thick were sliced along two mutually perpendicular directions from the boundary of the implantation site. Furthermore, the drug concentration of the liver sections were analyzed using the validated UPLC-MS/MS method. We determined the average DOX concentration within 2 cm of the boundary of implantation site.

### 2.4. Determination of DOX in liver tissues

#### 2.4.1. Sample preparation for UPLC-MS/MS determination

To pretreat the tissue samples, defined amount of liver tissue was accurately weighed (0.1 g) and homogenized in ultra-pure water containing 0.1% (v/v) formic acid for 3 min using tissue homogenizer. After being centrifuged at 8000 rpm for 10 min, an aliquot of 100 μl tissue homogenate was mixed with 20 μl Glibenclamide (internal standard, IS) working solution (200 ng/ml) by vortex-mixing for 30 s, followed by addition of 300 μl 0.1% (v/v) formic acid-acetonirile and again vortexed for 5 min, centrifuged at 12000 rpm for 10 min. After centrifugation, 5 μl of the supernatant was injected for analysis.

#### 2.4.2. Instrument conditions

Chromatographic separation was performed using the Waters ACQUITY ultra- performance liquid chromatography (WatersCorp., MA, USA) and the mass analysis was carried out with the Quattro-Premiere system (Waters Corp., MA, USA). Data were acquired and processed by the MassLynx software (version 4.1). The column used was Acquity UPLC BEH C18 (Waters Corp., 50 mm × 2.1 mm, 1.7 µm particles).

Ultra-pure water (A) and acetonitrile (B), both containing 0.1% (v/v) formic acid, were used for separating the analytes on the BEH C18 column at a flow rate of 0.2 mL/min under gradient elution: 0 min, 5% B; 0.5 min, 5% B; 1.2 min, 95% B; 2.2 min, 95% B; 3.0 min, 5% B; 4.0 min, 5% B. The column oven temperature was set at 37 °C and the autosampler temperature was maintained at 10 °C.

The optimal conditions for analysis were as follows: capillary voltage 2.7 kV, cone voltage 20 V, desolvation gas (nitrogen) 350 °C and 650.0 L/hour, and collision energy 15 eV. Detection was performed using positive electrospray ionization (ESI) source via multiple reaction monitoring (MRM) mode. The MRM transitions were m/z 544.1 → 397.2 for DOX and m/z 494.1 → 368.9 for IS.

#### 2.4.3. Method validation

The method for detecting DOX in liver tissues was fully validated in accordance with US Food and Drug Administration (FDA) guidelines and European Medicines Agency (EMEA) guidelines for bioanalytical method validation (U.S. Department of Health and Human Services, Food and Drug Administration (FDA), Center for Drug Evaluation and Research (CDER), 2018; European Medicines Agency, Committee for Medicinal Products for Human Use, 2011).The method validation was performed in terms of selectivity, calibration curve, accuracy, precision, extraction recovery, matrix effect, carry-over effect, stability and dilution test.

### 2.5. Data analysis

All data were expressed as mean ± SEM and analyzed using GraphPad Prism version 7.0 software (San Diego, CA). One-way ANOVA of Tukey’s multiple comparison tests was used to compare the mean of all experimental groups. The *p* value < .05 was considered as statistical significance.

## 3. Results

### 3.1. Preparation of DOX-loaded implants

The DOX-loaded implants were prepared by blending and melting the mixture of DOX, PLGA and PEG 4000. The implants were further molded into cylinder with the average diameter of (0.86 ± 0.01) mm and length of (4.00 ± 0.23 mm) (Supplementary Figure S1). Moreover, the average weight of the implants was (2.88 ± 0.29) mg (*n* = 10).

### 3.2. Content uniformity

Content uniformity testing is a pharmaceutical analysis parameter for the quality control of solid dosage. To determine the content uniformity of the DOX-loaded implants, ten implants were selected and tested the drug content by HPLC complying with the method in the Chinese Pharmacopeia (China Pharmacopoeia Committee, 2015). As shown in Supplementary Table S1, the mean value of actual drug content of the tested implants was assayed to be (25.02 ± 0.21) %. The acceptance value (AV) was calculated to evaluate drug content uniformity according to the formula AV = |100 – $\bar{\text{X}}$| + 2.2 S. In the work, $\bar{\text{X}}$ was 100.096 and S was 0.84, the AV of content uniformity was calculated to be 1.944 which was significantly lower than the maximum allowed acceptance value, indicating that the content uniformity of the DOX-loaded implants met the requirements of the Chinese Pharmacopeia.

### 3.3. Micromorphology of DOX-loaded implants

**Figure 1.**
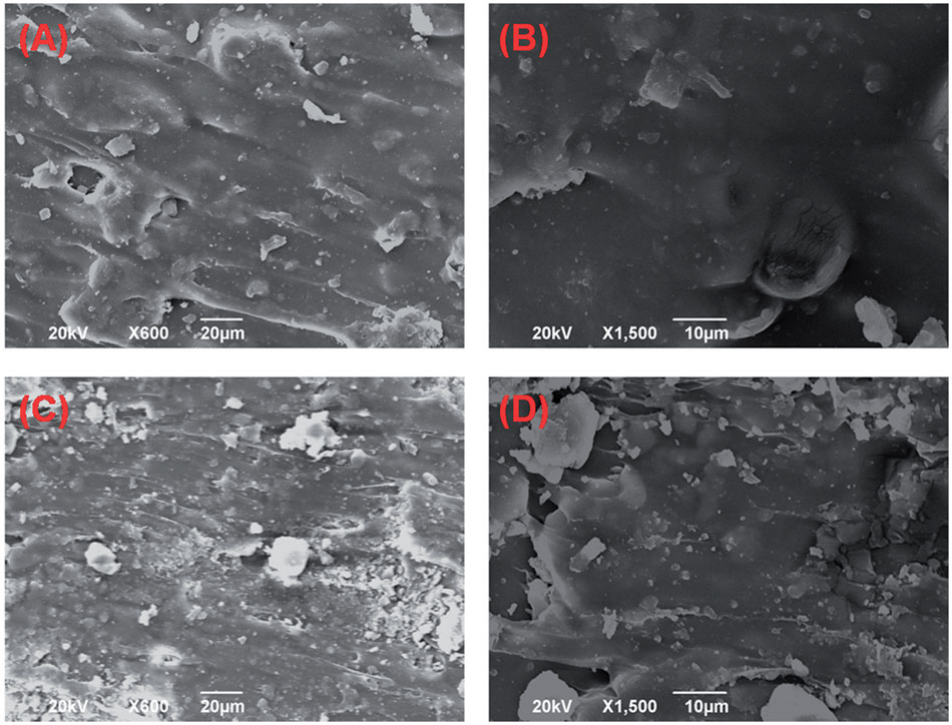
SEM pictures of the DOX-loaded implants. (A) External surface of the implant (magnification ×600). (B) External surface of the implant (magnification ×1500). (C) Cross-section of the implant (magnification ×600). (D) Cross-section of the implant (magnification ×1500).

SEM was used to evaluate the microstructure of the DOX-loaded implants. The external surface of the implant was found to be smooth and homogenous (Figure 1(A,B)). The cross-section of the implant was shown in Figure 1(C,D). We found that the micromorphology of the internal surface was a little rough but still homogenous without obvious pores or channels. The SEM images of the DOX-loaded implants revealed that the drug was uniformly distributed in the implants.

### 3.4. In vitro and in vivo drug release

**Figure 2.**
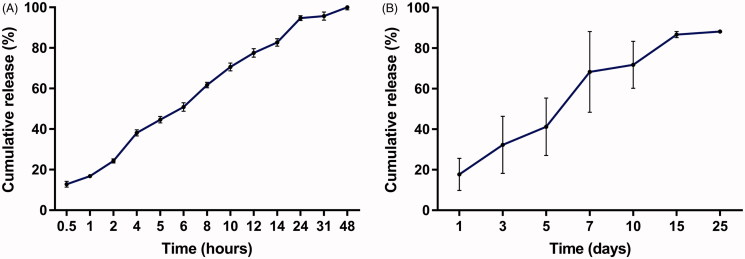
Release profiles of the DOX-loaded implants. (A) *In vitro* release profile of the implant. (B) *In vivo* release profile of the implant.

The *in vitro* cumulative release test was carried out using trimethylol aminomethane buffer as the release medium. The *in vitro* release profile was depicted in Figure 2(A). The implants released approximately 12.8% of DOX in the first 0.5 h. The mean cumulative release percentage was 50.9% within 6 h. We observed 94.7% of DOX released from the implants in 24 h. Furthermore, the cumulative drug release approximately reached 100% in 48 h.

To gain the information of the *in vivo* release profile, one DOX-loaded implant was implanted into the liver of Wistar rats. Then the implant was collected at the predetermined time points. As shown in Figure 2(B), the implant released 17.7% of drug on day 1 and 41.2% of drug within 5 days. The mean cumulative release reached 71.8% on day 10. Subsequently, the drug release slowed down and the cumulative drug release was approximately 90% within 25 days.

### 3.5. In vivo degradation study

The *in vivo* degradation of the implants was analyzed over 7 weeks. The result was shown in Supplementary Figure S2. We observed 9.6% weight loss of the implant within the first week. Then the the blank implants degradated almost at a constant rate. Furthermore, the samples lost ∼81% of their original weight in 7 weeks. It is difficult to retrieve the residual blank implants at the 8th week post implantation.

### 3.6. DSC analysis

**Figure 3.**
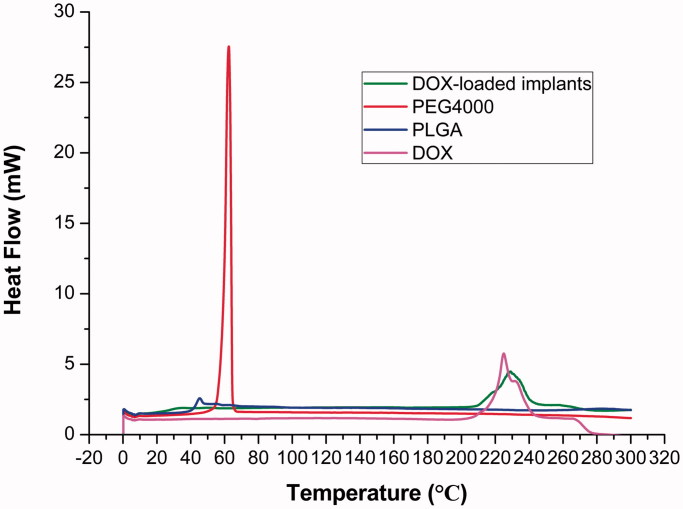
DSC curves of DOX, PLGA, PEG 4000 and DOX-loaded implants.

The thermal behaviors of DOX, PLGA, PEG 4000 and DOX-loaded implants were analyzed by DSC. As shown in Figure 3, the curve of pure DOX showed a broad endothermic peak in the temperature between 210 °C and 240 °C. The DSC traces of PLGA and PEG 4000 exhibited a melting sharp endothermic peak centered at approximately 45 °C and 60 °C, respectively. Additionally, the DOX-loaded implants exhibited a thermal behavior similar to that identified for pure DOX and the polymers.

### 3.7. FTIR analysis

**Figure 4.**
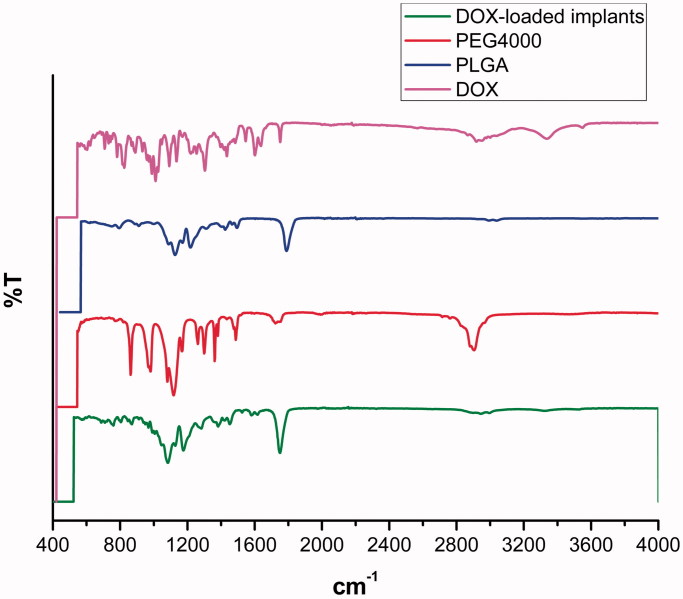
FTIR spectra of DOX, PLGA, PEG 4000 and DOX-loaded implants.

The FTIR spectra of DOX, PLGA, PEG 4000 and DOX-loaded implants were shown in Figure 4. In the FTIR spectrum of DOX, the peaks at 3552 cm^−1^, 3328 cm^−1^, 2944 cm^−1^ and 1754 cm^−1^ were observed. Typical infrared absorption bands observed in PLGA and PEG 4000 were detected in the spectra of DOX-loaded implants.

### 3.8. Local drug penetration of DOX-loaded implants

#### 3.8.1. UPLC-MS/MS method for detection DOX in liver

In this study, we established an UPLC-MS/MS method to quantitate DOX in liver tissues of minipigs. Then the established method was fully validated in compliance with FDA guidelines and EMEA guidelines for bioanalytical method validation. The results showed that the method met the acceptance criteria of the guidelines, indicating that the UPLC-MS/MS method for detecting DOX is accurate and reliable (data not shown).

#### 3.8.2. Local drug penetration of DOX-loaded implants in liver

**Figure 5.**
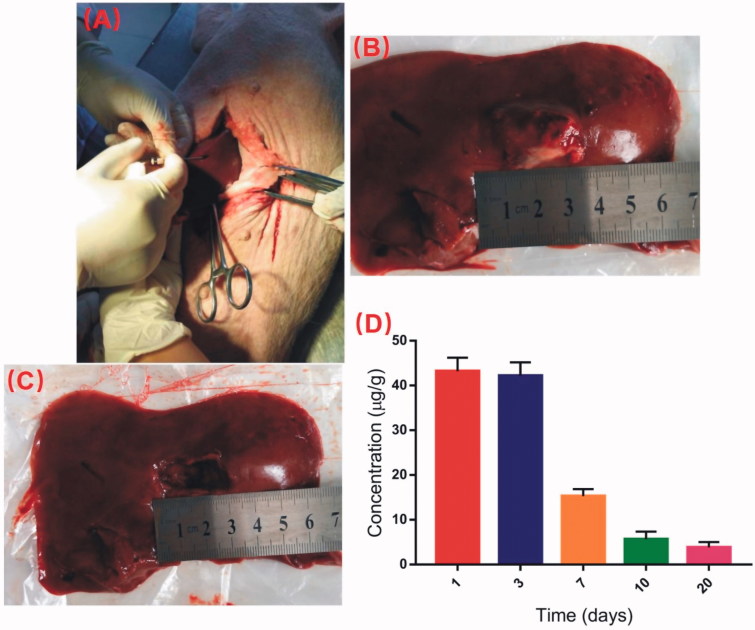
Detection of DOX concentration in liver at the implantation site. (A) The DOX-loaded implants were implanted into the left lobe of liver. (B) The picture of the implantation site after intrahepatic implantation of the DOX-loaded implants. (C) The drug residues were removed before detecting the DOX concentration in liver tissues. (D) Concentration of DOX in liver tissues at the implantation site.

The minipigs were anesthetized and received abdominal surgery under sterile conditions. The DOX-loaded implants were inserted into the left lobe of the liver using the modified 17-gauge trochar at the dose of 2.3 mg/kg (Figure 5(A)).

The drug residues in the implantation site were removed at predetermined time intervals after intrahepatic implantation of DOX-loaded implants (Figure 5(B,C)). Then the DOX concentration in liver tissues were detected using UPLC-MS/MS method. As shown in Figure 5(D), the mean concentration of DOX at the implantation site of liver reached 43.2 μg/g on the first day post implantation. Moreover, the drug concentraion was detected as high as 42.2 μg/g on Day 3. The DOX concentration in liver tissues declined obviously from Day 7. The liver concentraion of DOX was 3.8 ug/g on Day 20 after intrahepatic implantation of the implants.

**Figure 6.**
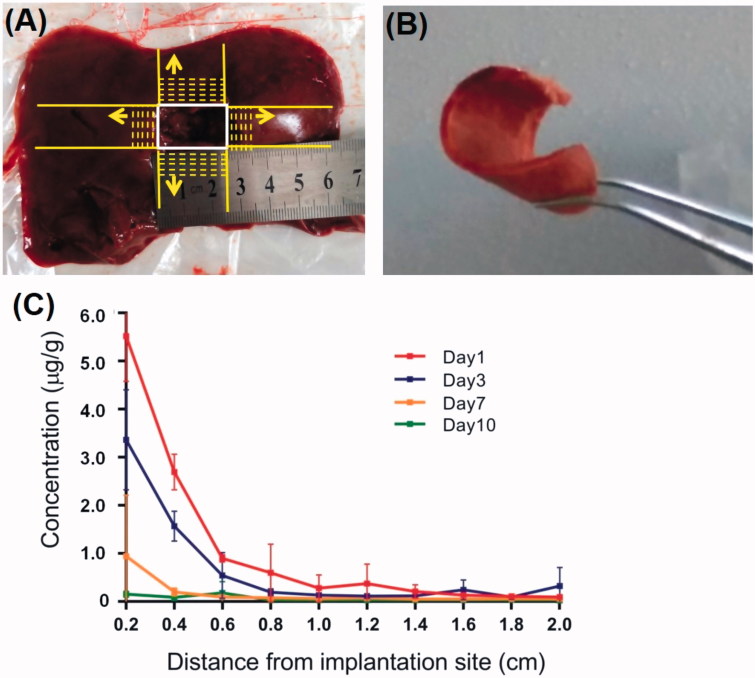
Local penetration of DOX from DOX-loaded implants. (A) Serial frozen liver tissue sections were sliced in two mutually perpendicular directions from the boundary of the implantation site. (B) The frozen liver tissue section of 2 mm thick. (C) Concentration of DOX in liver tissues around the implantation site.

To observe the local drug penetration of the DOX-loaded implants, serial frozen liver sections of 2 mm thick were sliced along two mutually perpendicular directions from the boundary of the implantation site (Figure 6(A,B)). The concentration of DOX in liver tisue sections were detected using the UPLC-MS/MS method on day 1, 3, 7 and 10 post implantation. As shown in Figure 6(C), the mean concentration of DOX at a distance of 2 mm from the boundary of implantation site was (5.5 ± 0.9) μg/g on the first day and (3.3 ± 1.0) μg/g on Day 3. The concentration of DOX around the implantation site decreased obviously with the increase of distance and the extension of time within 1 cm of the margin of the implantation site.

## 4. Discussion

Local chemotherapy provides high drug concentration at the tumor site for a prolonged period, therefore it is considered to be effective for lowering post-surgical recurrence. Local tumor recurrence remains a major clinical problem following surgical treatment for most cancers such as hepatocellular carcinoma (HCC). It has been reported that recurrence of HCC occurs in 50–80% of the patients within 2–5 years after resection (Liu et al., 2015). Post-surgical local chemotherapy would be a solution to reduce recurrence near the surgical margin. In the study, we prepared the DOX-loaded implants and characterized the implants regarding drug content uniformity, micromorphology, drug release profiles and drug-excipient compatibility. We then further described the local drug penetration after intrahepatic implantation of the implants. We believe that our research data can provide valuable information for future post-surgical application of the DOX-loaded implants.

The DOX-loaded implants were prepared as cylinder by a simple and reproducible melt-molding method. PLGA was the main excipient used in the fabrication of the DOX-loaded implants. PLGA is a copolymer of polylactic acid and polyglycolic acid which has been widely used in drug delivery systems, tissue engineering, and medical and surgical devices. PLGA is approved by the FDA in the US for therapeutic applications because of its biodegradability, biocompatibility, and sustained-release properties. Additionally, PLGA has been widely used as biodegradable, nontoxic and non-immunogenic polymer for the development of controlled drug delivery systems (Kapoor et al., 2015; Xu et al., 2017). PEG 4000 was the other excipient of the implants which was characterized by low melting point, low toxicity, good compatibility and hydrophilicity. PEG has been widely used in the drug delivery systems. Furthermore, addition of PEG can facilitate the dissolution and increase the release of drug from the implants (Gao et al., 2017).

Content uniformity testing is a pharmaceutical analysis parameter for the quality control of solid dosage. In this work, the acceptance value for content uniformity of the DOX-loaded implants was calculated to be 1.944 which met the requirements of the Chinese Pharmacopeia. The result suggested that DOX and the excipients were sufficiently mixed in the fabrication process and DOX dispersed uniformly in the polymers.

The micromorphology of the DOX-loaded implants showed that the external surface and cross-section of the implants were homogenous without obvious pores and channels. The results indicated that the fabrication procedure of the implants yielded a uniform distribution of DOX into the polymeric matrix.

The release profile of DOX-loaded implants demonstrated a burst effect followed by sustained-release both *in vitro* and *in vivo*. The initial fast release may be due to fast dissolution and diffusion of DOX accumulated on the surface of the implants. The rate of drug release from the drug delivery systems are affected by the environmental conditions, physicochemical properties of the polymer, drug characteristics, the drug loadings, etc. (Solano et al., 2013). In the study, we recorded the faster release of DOX from the implants *in vitro* than *in vivo*, this phenomenon could be due to the different physiological environment for drug release. It is generally accepted that the optimal drug release profile of the implants should be characterized by the ability to release drug to a large volume and maintain the therapeutic concentration for a prolonged period (Weinberg et al., 2008). In the present study, the DOX-loaded implants released a large amount of the drug firstly so that it can rise to the therapeutic concentration rapidly, then the following sustained-release of drug could maintain the therapeutic concentration for an extended time. The DOX-loaded implants exhibited more adequate release of DOX and longer duration of *in vivo* drug release compared with the reported DOX-loaded solid formulation (Ortiz et al., 2007; Weinberg et al., 2007; Solorio et al., 2016; Kefayat & Vaezifar, 2019). In addition, the release profile of the DOX-loaded implants can maximize the treatment success of the implants.

DSC is the most common thermal analysis technique used to examine the physical properties of implants. Our DSC thermogram of DOX-loaded implants showed all endothermic events corresponding to DOX, PLGA, and PEG 4000. We observed the endothermic peak of DOX in the range of 210–240 °C, centered at about 225 °C, which was related to the melting of the drug. The melting peak of PLGA and PEG 4000 occurred at a low temperature. The low melting point of the polymers is essential for the implant formulation in order to avoid the instability of compounds (Gupta et al., 2012). We did not find new endothermic or exothermic peak in the DSC curve of DOX-loaded implants, indicating that there was no chemical interaction between DOX and excipients.

The FTIR analysis was used to determine drug-excipient interaction at the level of functional groups (Rudra et al., 2010). DOX has different reactive functional groups, such as free NH_2_ group, OH group, H, and CO (Rudra et al., 2010). For FTIR spectra of DOX, the peak at 3552 cm^−1^ was attributed to N-H symmetric stretching. Additionally, the C-H stretching vibration at 2944 cm^−1^ and the peak at 3328 cm^−1^ confirm the presence of DOX in the complex (Neacșu, 2018). Typical infrared absorption bands of the functional groups visualized in pure DOX, PLGA and PEG 4000 were also observed in the FTIR spectra of the DOX-loaded implants. Moreover, we did not observe new bands in the FTIR spectra of the DOX-loaded implants. Therefore, the FTIR data were in agreement with the DSC analysis results, supporting that no chemical interactions occurred between the drug and the polymers during the manufacturing process of the implants.

Hepatectomy is an effective treatment for liver cancers. However, the 5-year recurrence rate of 50–70% after surgery has been reported. The postoperative recurrence frequently undermines the long-term survival outcomes of hepatectomy (Peng et al., 2019). Several novel implantable drug-delivery strategies have been reported to prevent locoregional recurrence, such as polymer films, wafers and hydrogels (Mahvi et al., 2018). In this study, we prepared the PLGA based DOX-loaded implants and observed the local penetration of the implants in the liver of minipigs. The doses of DOX-loaded implants given to the minipigs were calculated according to the clinically used dosage of DOX (60 mg/m^2^) (Huang et al., 2004; Park et al., 2006). In addition, the pre-experiment showed that the minipigs tolerated well to the dosage of DOX-loaded implants (2.3 mg/kg). To determine the concentration of DOX in liver tissues, we established an accurate and reliable UPLC-MS/MS method. The results revealed that the DOX concentration could achieve high level in the implantation site after removal of the residues of the implants. Though the concentration of DOX declined obviously from Day 7, we still observed that the DOX concentration remained high for a prolonged time. It has been reported that residual microscopic tumor cells at the surgical margin have been identified postoperatively in 39% of patients following “curative” wedge resection (Zhang et al., 2014). Locoregional implantation of DOX-loaded implants has the potential be an alternative option for the prevention of local recurrence within the tumor bed or near the surgical margin.

The DOX concentrations around the implantation site were also detected. We found that the drug concentration reduced sharply over very short distance from the boundary of the implantation site. The result was similar to the previous literature (Weinberg et al., 2007). The local dispersion of drug is governed by physiological transport principles (Hwang et al., 2001). An important transport mode of drug in tissues is usually diffusion by the concentration gradients. Furthermore, local anatomy and microenvironment of the tissue are potential barriers to local drug transport (Weiser & Saltzman, 2014). It is known that the liver is rich in blood vessels. In the study, the limited penetration of DOX in liver may be explained that DOX was trans-membrane transported into the local capillaries and eliminated via the capillary blood flow. Thus, the DOX-loaded implants may not be able to effectively kill the remaining cancer cells far away from the surgical margin because of its inadequate distribution distance.

## 5. Conclusion

In the present study, we prepared the DOX-loaded implants with a melt-molding technique. The resutls of drug content uniformity indicated that DOX was homogeneously dispersed in the polymeric matrix. Both *in vitro* and *in vivo* release profiles of the implants were characterized by initial burst release followed by sustained-release of the DOX. The release profile of the DOX-loaded implants will be able to maximize the treatment success of the implants. The DSC and FTIR data indicated good compatibility between the DOX and the excipients. Results from local penetration of DOX after intrahepatic implantation of the DOX-loaded implants demonstrated that the drug concentration at the implantation site could remain at a high levle for a prolonged time. Locoregional implantation of DOX-loaded implants has the potential to be an alternative option for the prevention of local recurrence within the tumor bed or near the surgical margin.

## Supplementary Material

Supplemental Material

Supplemental Material

Supplemental Material
