# Supplementary material for: Local penetration of doxorubicin via intrahepatic implantation of PLGA based doxorubicin-loaded implants
Source: Drug Deliv. 2019 Nov 6;26(1):1049–57. doi: 10.1080/10717544.2019.1676842 (PMC6844384; doi:10.1080/10717544.2019.1676842)
Supplement: Supplemental Material [file IDRD_A_1676842_SM3507.docx]

Table S1.

The drug content of DOX-loaded implants

| DOX-loaded implants | Label claim of drug  (%) | Actual drug content  (%) | Relative content |
| --- | --- | --- | --- |
| 1 | 25.00 | 25.44 | 101.76 |
| 2 | 25.00 | 24.93 | 99.72 |
| 3 | 25.00 | 25.16 | 100.64 |
| 4 | 25.00 | 25.14 | 100.56 |
| 5 | 25.00 | 25.04 | 100.16 |
| 6 | 25.00 | 24.99 | 99.96 |
| 7 | 25.00 | 24.65 | 98.60 |
| 8 | 25.00 | 25.10 | 100.40 |
| 9 | 25.00 | 24.95 | 99.80 |
| 10 | 25.00 | 24.84 | 99.36 |
