# Supplementary figures and images for: Local penetration of doxorubicin via intrahepatic implantation of PLGA based doxorubicin-loaded implants
Source: Drug Deliv. 2019 Nov 6;26(1):1049–57. doi: 10.1080/10717544.2019.1676842 (PMC6844384; doi:10.1080/10717544.2019.1676842)

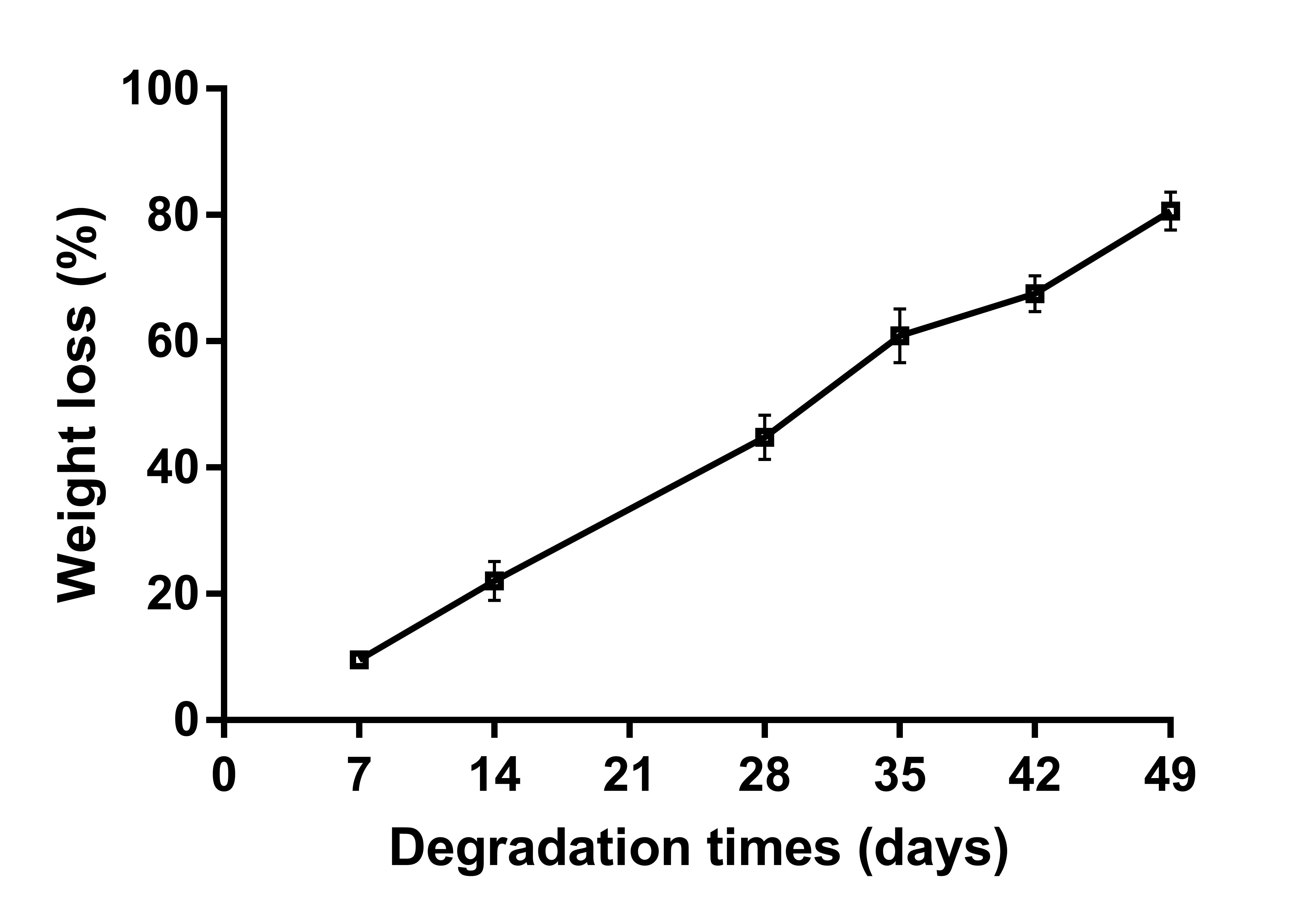

Supplement: Supplemental Material [file IDRD_A_1676842_SM3506.jpg]

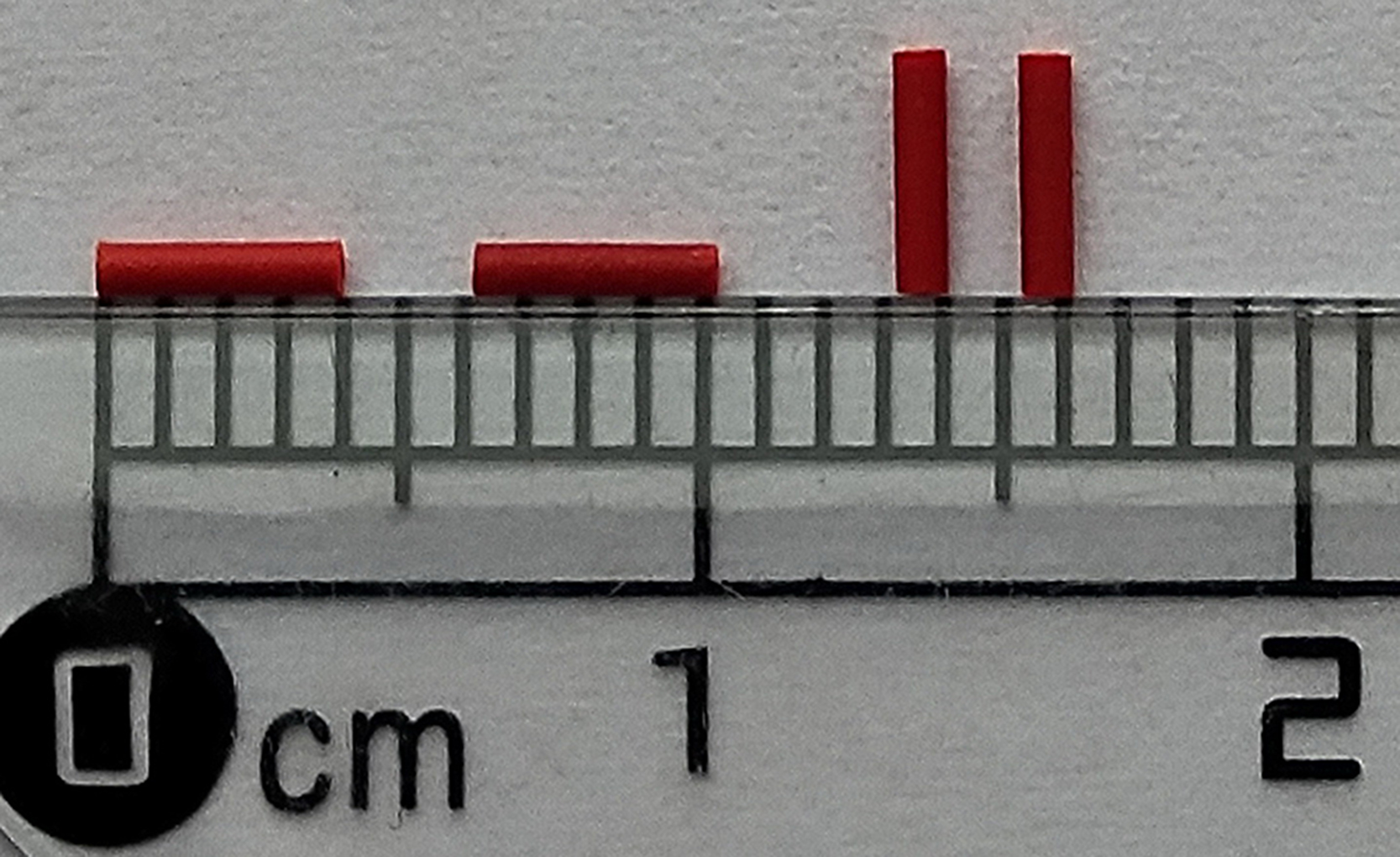

Supplement: Supplemental Material [file IDRD_A_1676842_SM3505.jpg]
